# A Distal Femoral Salter-Harris IV Fracture Secondary to a Motocross Accident: A Case Report

**DOI:** 10.7759/cureus.38679

**Published:** 2023-05-07

**Authors:** Christiaan Van Nispen, Rachel E Bridwell, Joel J Fernandes, Brit Long

**Affiliations:** 1 Emergency Medicine, San Antonio Uniformed Services Health Education Consortium, Fort Sam Houston, USA; 2 Emergency Medicine, Madigan Army Medical Center, Joint Base Lewis-McChord, USA; 3 Special Operations Combat Paramedicine, Army Special Operations Aviation Command, Fort Bragg, USA

**Keywords:** type iv, salter-harris, physeal injury, femur, fracture, motocross

## Abstract

High-grade physeal fractures, such as Salter-Harris types III, IV, and V fractures, are rare pediatric injuries observed disproportionately in teenage males. Such fractures are at high risk for complications such as growth retardation and arrest, arthrofibrosis, and post-traumatic arthritis. Consultation with the orthopedic specialist is imperative to ensure appropriate imaging, management, and potential transfer to a pediatric specialty hospital. The authors present a case of a 15-year-old male who sustained a Salter-Harris IV fracture of the distal femur extending from the intercondylar notch to the metadiaphysis from a motocross accident.

## Introduction

Comprising 1-6% of all physeal injuries and less than 1% of all fractures in the pediatric population, fractures involving the distal femoral epiphysis are uncommon causes for presentation to the emergency department [1-3]. The population most associated with distal femoral physeal fractures is adolescent males, although such fractures can occur in any skeletally immature patient [4]. The most common causes include high-energy injuries such as sporting activities and motor vehicle accidents [5]. Physeal fractures increase the risk for premature physeal closure leading to limb length discrepancies, particularly when undertreated. This is of particular concern with the distal femoral physis, which is the fastest-growing physis in the body, responsible for 35% of longitudinal growth [4]. When physeal fractures involve the joint, as in Salter-Harris types III and IV fractures, additional complications, such as arthrofibrosis and post-traumatic arthritis, can ensue [4]. According to one study, 39% of such fractures are missed or insufficiently characterized by the initial treating clinician [4]. Given the risk of morbidity, emergency clinicians must be able to identify and treat this patient population promptly and correctly. The authors present the case of a 15-year-old male who sustained a Salter-Harris type IV fracture of the distal femur extending from the intercondylar notch to the metadiaphysis due to a motocross accident.

## Case presentation

A 15-year-old male presented to a community hospital emergency department (ED) for evaluation of left knee pain and difficulty with weight bearing of the left lower extremity after an accident during a motocross race. The patient stated that he was riding a motocross bike at 25 to 30 miles per hour around a corner when to avoid a competitor, he planted his left foot onto the ground causing hyperflexion of his left knee joint.

Initial vital signs were temperature 98.3℉, heart rate 73 beats per minute, respiratory rate 17 breaths per minute, blood pressure 110/65 mmHg, and oxygen saturation 98% on room air. Physical examination revealed edema and tenderness localized to the left distal femur and patella, with active extension at the left knee limited by pain. The remainder of the physical examination was unremarkable.

A plain radiograph of the left knee demonstrated an intra-articular fracture of the distal femur with an associated large effusion of the left knee joint.

**Figure 1:**
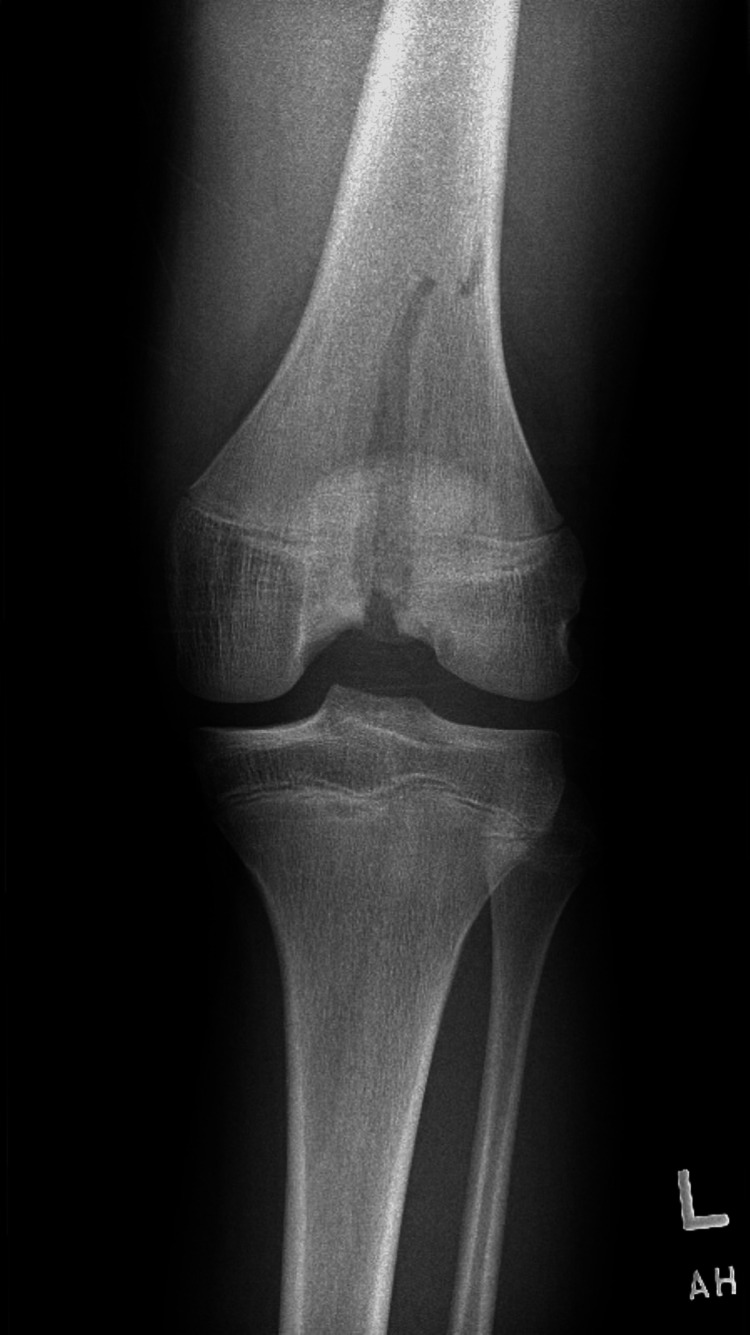
Left knee radiograph, anteroposterior view

Orthopedic consultation recommended computed tomography (CT) with 3D reconstruction for operative planning and magnetic resonance imaging (MRI) of the left knee to assess for ligamentous injury. The CT redemonstrated a fracture of the distal femur extending from the distal femoral epiphysis at the intercondylar notch to the lateral aspect of the distal femoral metadiaphysis, with up to 7 mm of separation at the fracture site. While there was no injury to the cruciate and collateral ligaments or either meniscus, the MRI demonstrated a mild partial interstitial tear of the distal quadriceps, a large hemarthrosis, and a large hematoma of the popliteal fossa.

**Figure 2:**
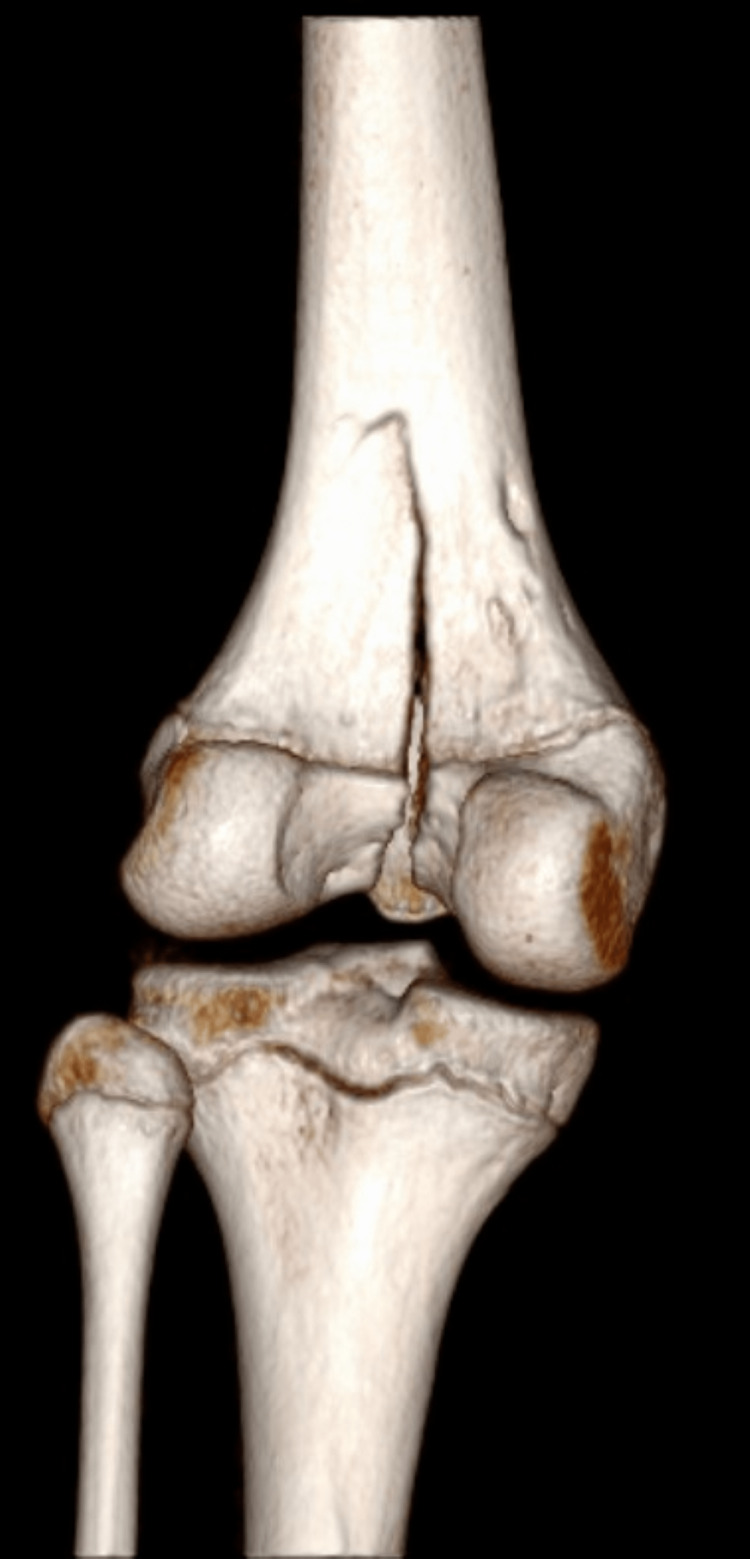
CT with 3D reconstruction of the left knee, posterior view

**Figure 3:**
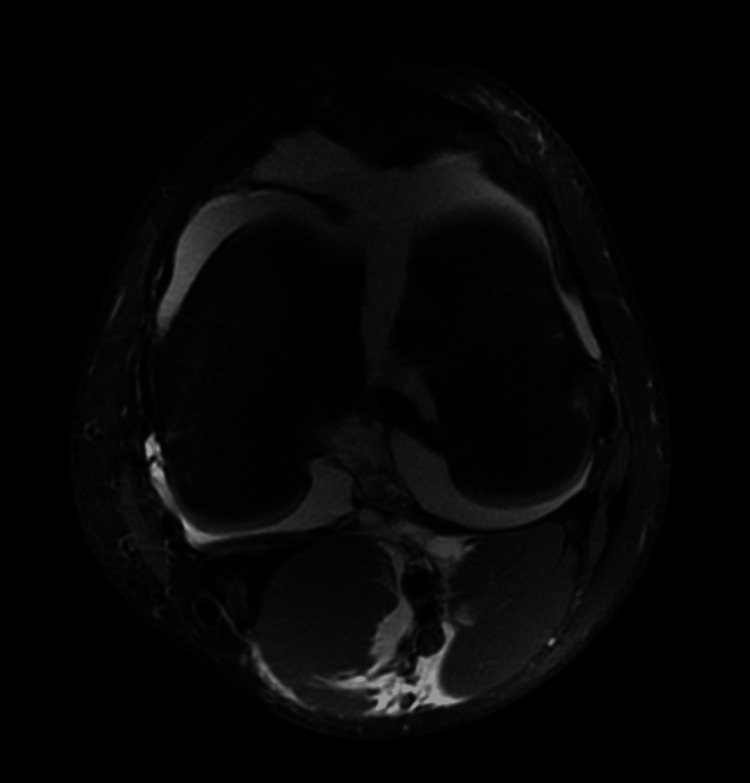
MRI of the left knee, transverse view

The patient was admitted to the pediatrics service preoperatively and underwent left knee arthroscopy and left femur open reduction and internal fixation with the placement of two cannulated screws. The patient recovered well; he was discharged on postoperative day 1, returned to full weight-bearing at three months postoperatively, and to sporting activities, including Motocross at five months postoperatively.

## Discussion

Salter-Harris type IV fractures are uncommon pediatric injuries that require prompt recognition in order to ensure proper management and reduce complications [1]. More common in males than females, Salter-Harris type IV fractures comprise just 10% of all physeal fractures and most commonly affect the upper extremities [1]. Emergency clinicians should maintain a high index of suspicion for such fractures when pediatric patients present with pain, decreased range of motion, or difficult weight-bearing localizing to a joint after a high-energy mechanism [1]. As Salter-Harris fracture types III and higher typically require prompt operative intervention, consultation with an orthopedic specialist in the emergency department is essential [1].

In this case, plain radiographs clearly demonstrated a Salter-Harris type IV fracture; however, emergency clinicians should consider advanced imaging, as plain radiographs can underestimate physeal displacement [6]. Indeed, this injury pattern is frequently misdiagnosed by the initial treating clinician [4]. A small retrospective review of pediatric patients with distal femur Salter-Harris type III fractures demonstrated a statistically significant difference in the measured displacement on plain radiograph versus CT or MRI, with the treatment of 29% of patients changing as a result, as underestimation of physeal displacement can lead to incorrect management recommendations from orthopedic specialists [6-7]. Jaramillo et al. reported that MRI alone can sufficiently determine which physeal zones are involved, which avoids exposure to ionizing radiation [8].

Advanced imaging with CT and MRI in the ED played an important role in the evaluation and management of this patient. While the CT allows improved characterization of osseous injuries compared to plain radiographs, MRI adds the additional benefit of identifying concomitant soft tissue injuries. Both modalities assist with operative planning. The ability to obtain CT and/or MRI imaging during an emergency department course will vary by institution and clinical context. The choice in advanced imaging should be made in consultation with the orthopedic specialist. Emergency clinicians will frequently be the first to encounter these patients in the clinical setting, making it imperative that correct imaging is obtained to ensure appropriate management. Further study is necessary to determine the merits of obtaining CT versus MRI or both in the context of high-grade physeal fractures.

## Conclusions

Salter-Harris type IV fractures of the distal femur are uncommon pediatric injuries, indicating a high-energy mechanism. Imaging is key to proper assessment. In addition to radiographs, cross-sectional imaging is necessary to fully characterize the fracture, identify concomitant soft tissue injuries, and guide operative intervention. Prompt recognition and consultation are crucial to mitigate the high complication rate, including growth retardation and arrest, arthrofibrosis, and post-traumatic arthritis. Emergency clinicians must consider this rare fracture pattern and obtain a consultation with an orthopedic specialist to best care for this injury and minimize morbidity.

## References

[1] Singh A, Mahajan P, Ruffin J, Galwankar S, Kirkland C (2021). Approach to suspected physeal fractures in the emergency department. J Emerg Trauma Shock.

[2] Cepela DJ, Tartaglione JP, Dooley TP, Patel PN (2016). Classifications in brief: Salter-Harris classification of pediatric physeal fractures. Clin Orthop Relat Res.

[3] Aydin A, Topal M, Tuncer K, Senocak E (2012). Salter-Harris type III and type IV combined fracture of the distal femoral epiphysis: a case report. Case Rep Med.

[4] Pennock AT, Ellis HB, Willimon SC, Wyatt C, Broida SE, Dennis MM, Bastrom T (2017). Intra-articular physeal fractures of the distal femur: a frequently missed diagnosis in adolescent athletes. Orthop J Sports Med.

[5] Beaty JH, Kumar A (1994). Fractures about the knee in children. J Bone Joint Surg Am.

[6] Mills L, Zeppieri G Jr (2019). Salter-Harris type III fracture in a football player. J Orthop Sports Phys Ther.

[7] Lippert WC, Owens RF, Wall EJ (2010). Salter-Harris type III fractures of the distal femur: plain radiographs can be deceptive. J Pediatr Orthop.

[8] Jaramillo D, Kammen BF, Shapiro F (2000). Cartilaginous path of physeal fracture-separations: evaluation with MR imaging--an experimental study with histologic correlation in rabbits. Radiology.

